# Indistinguishability of temporally separated pairwise two-photon state of thermal photons in Franson-type interferometry

**DOI:** 10.1038/s41598-022-09516-y

**Published:** 2022-03-31

**Authors:** Jiho Park, Heonoh Kim, Han Seb Moon

**Affiliations:** Department of Physics, Pusan National University, Geumjeong-Gu, Busan, 46241 South Korea

**Keywords:** Quantum optics, Quantum optics, Single photons and quantum effects

## Abstract

The phenomenon of Franson interference with time–energy entangled photon pairs beyond the single-photon coherence length observed upon nonlocal measurement at two space-like separated locations is of particular research interest. Herein, we determine the coherence length of temporally separated pairwise two-photon (TSPT) states of thermal photons emitted from a warm atomic ensemble in Franson-type interferometry, with the setup consisting of two spatially separated unbalanced Michelson interferometers beyond the coherence length of a thermal photon. Using a novel method of square-modulated thermal photons, we show that the sinusoidal Franson-type interference fringe of thermal photons is determined by the presence or absence of TSPT states (corresponding to the time delay between the long and short paths in Franson-type interferometry). We find that the indistinguishability of the TSPT state in the Franson-type interference is independent of the temporal separation of the thermal photons in the TSPT states.

## Introduction

A thorough understanding of the phenomenon of interference is essential to investigate the nature of light from the viewpoints of both classical and quantum physics. In this regard, the coherence of light is used to describe the correlation between the phases of two lights. In particular, interference can be observed as a function of the path-length difference in an interferometer within the coherence length of light. To suitably define the phase relation, a light should be separated and superposed. From the photon perspective, a single photon can only interfere with itself^1^, and the observed interference pattern in this case can be described in terms of the quantum interference of the photon wavefunction. The coherence of photons is related to the intrinsic indistinguishability of particle trajectories that give rise to the interference pattern^2^.

In this context, we consider Franson interference, which is a well-known second-order two-photon quantum interference phenomenon^3,4^. In particular, the two-photon coherence length of a time–energy entangled photon pair beyond the single-photon coherence length is a counterintuitive phenomenon^3–13^. To avoid single-photon interference in Franson interference, it is necessary that the path-length difference of the unbalanced interferometry setup is considerably longer than the coherence length of the one-photon state. Franson interference technique can determine the two-photon coherence length of an entangled photon pair^6^. The two-photon coherence length depends on the characteristics of the time–frequency entangled photon pair originating from various media^4–13^.

Interestingly, the second-order interference (SOI) of thermal light in two independent unbalanced interferometers beyond the photon coherence length has been theoretically and experimentally reported^14–18^. Although thermal photons are not energy–time entangled, this SOI beyond the coherence length in the Franson-type interferometry is a highly counterintuitive phenomenon^14,16^.

In this work, we introduce the coherence length in a SOI with thermal photons as the indistinguishability of the temporally separated pairwise two-photon (TSPT) state in Franson-type interference for the first time. In this study, we propose the novel method for investigation of the temporal waveforms of thermal photons in Franson-type interferometry with and without the contribution of the TSPT states by using optical switching. We show that the SOI of the thermal photons in Franson-type interferometry is related to the intrinsic indistinguishability of the TSPT states of thermal photons, which is independent of the time delay between the temporally separated photons.

## Indistinguishable events of temporally separated pairwise two-photon states

**Figure 1:**
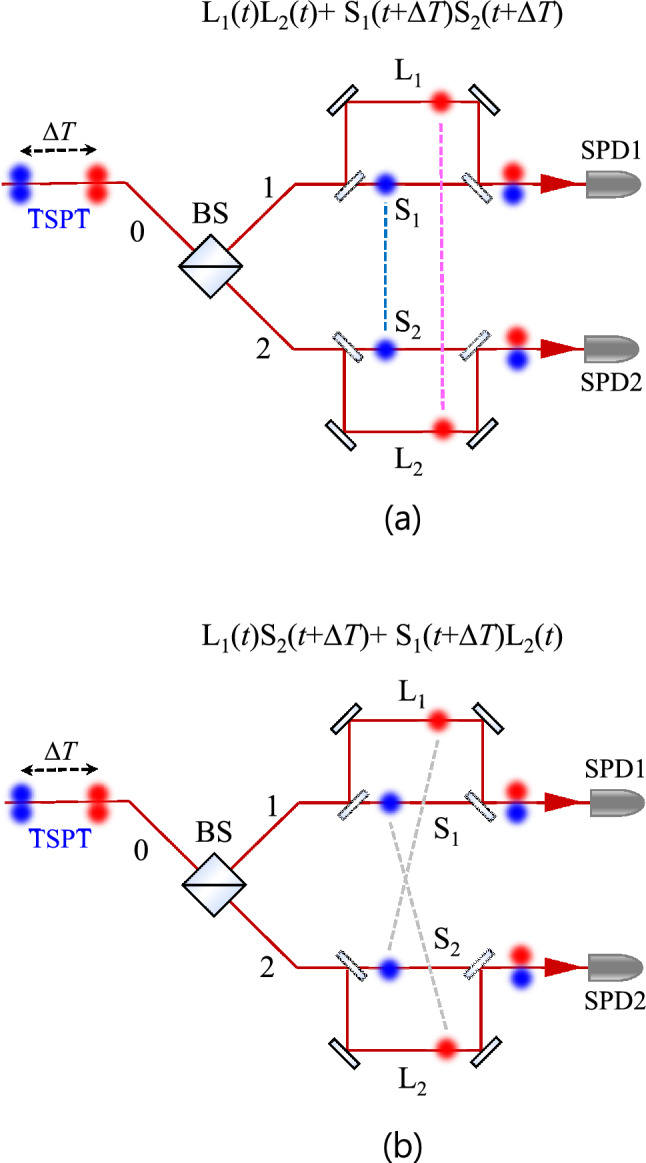
Indistinguishable events of temporally separated pairwise two-photon states in Franson-type interferometry. Coincidence events of the two detectors at τ = 0 in cases of (**a**) short-short (S_1_S_2_) and long-long (L_1_L_2_) paths and (**b**) long-short (L_1_S_2_) and short-long (S_1_L_2_) paths.

Here, we can define the coherence length of a two-photon state in a Franson-type interferometry as maximum path-length difference for possible interference. We first briefly describe the indistinguishable events of the CW-mode thermal light in Franson-type interferometry. The optical path-length difference between the long (L_*i*_) and short (S_*i*_) paths is considerably longer than the coherence length of the thermal light, and in the abovementioned notation, the subscripts represent the two spatial modes of the interferometer arms corresponding to the two output ports (1, 2) of the beam splitter (BS). The cases in Fig. 1a depict the phase-independent Hanbury Brown and Twiss (HBT) experiments corresponding to the second-order self-correlation function. Unlike the original Franson interference with entangled photon pairs, the indistinguishability of the two-photon state between the short-short (S_1_S_2_) and long-long (L_1_L_2_) paths in Fig. 1a affords no interference, because the two two-photon states of thermal photons are not correlated.

However, when we consider the time difference (Δ*T*) due to the path-length difference between the short and long paths, the two coincidentally detected photons of the long-short (L_1_S_2_) and short-long (S_1_L_2_) paths do not temporally overlap at the BS of the interferometer because the separation time can be considerably longer than the coherence time of the thermal light. However, the temporally separated thermal photons (red and blue circles) in the interferometer arms can be treated as a TSPT state (as indicated by the connecting gray dashed lines in Fig. 1b). The TSPT state with Δ*T* is described as^19^

1 $$ \left| \Psi \right\rangle |_{{{\text{TSPT}}}} = \frac{1}{\sqrt 2 }\left[ {a_{1}^{\dag } a_{2}^{\dag } (\Delta T) + a_{2}^{\dag } a_{1}^{\dag } (\Delta T)} \right]\left| {0,\,\,0} \right\rangle , $$

where $a_{i}^{\dag }$ denotes the photon creation operator, and the subscripts represent the two spatial modes of the interferometer arms according to the two output ports of the BS. The coincidence counts of the two detectors are related to the four cases of the S_1_S_2_, L_1_L_2_, L_1_S_2_, and S_1_L_2_ paths, as shown in Fig. 1a,b.

However, the two photons in a TSPT state with Δ*T* can be coincidently detected in the L_1_S_2_ and S_1_L_2_ paths, as shown in Fig. 1b. Although the TSPT states of the bunched photons are temporally separated beyond the coherence time of the CW-mode thermal light, the coincidence event of the TSPT state with Δ*T* via both the L_1_S_2_ and S_1_L_2_ paths is indistinguishable. The which-way information of the TSPT state of the thermal photons in the L_1_S_2_ and S_1_L_2_ paths of Franson-type interferometry is automatically erased. Furthermore, this interference is independent of the temporal separation of the TSPT state. Therefore, the coherence length of the TSPT state of the thermal photons in a Franson-type interferometry is unlimited.

The temporal waveform of the CW-mode bunched photons in Franson-type interferometry corresponds to the second-order correlation function $G^{(2)} (t_{1} ,\,t_{2} )$ in the two unbalanced interferometers, which can be expressed as^14^

2 $$ G^{(2)} (t_{1} ,\,t_{2} ) = G^{(1)} (t_{1} ,\,t_{1} )G^{(1)} (t_{2} ,\,t_{2} ) + \eta \left| {G^{(1)} (t_{1} ,\,t_{2} )} \right|^{2} . $$

here $G^{(1)} (t_{1} ,\,t_{2} )$ represents the first-order correlation function of the thermal photons, where $t_{i} \, (i = 1{\text{ and 2}})$ denotes the relative arrival times of the photons at SPD1 and SPD2, respectively. In addition, *η* represents the thermal fraction coefficient, including the time uncertainties of the both SPDs and their electronic time jitter.

The second term $\left| {G^{(1)} (t_{1} ,\,\,t_{2} )} \right|^{2}$ in Eq. (2) can be expressed as

3 $$ \left| {G^{(1)} (t_{1} ,\,\,t_{2} )} \right|^{2} = \left\{ {\begin{array}{*{20}l} {\left| {G^{(1)} (t_{L1} ,\,\,t_{S2} )} \right|^{2} } \hfill \\ { + \left| {G^{(1)} (t_{L1} ,\,\,t_{L2} ) + G^{(1)} (t_{S1} ,\,\,t_{S2} ) + G_{TSPT}^{(1)} (t_{L1} ,\,\,t_{S2} ) + G_{TSPT}^{(1)} (t_{S1} ,\,\,t_{L2} )} \right|^{2} } \hfill \\ { + \left| {G^{(1)} (t_{S1} ,\,\,t_{L2} )} \right|^{2} } \hfill \\ \end{array} } \right\}, $$

where $t_{i} \, (i = {\text{S}}_{1} ,{\text{ S}}_{2} ,{\text{ L}}_{1} ,{\text{ and L}}_{2} )$ denotes the relative arrival times of the photons at SPD1 and SPD2 via the short (S_1_, S_2_) and long (L_1_, L_2_) paths, respectively. In particular, $G_{TSPT}^{(1)}$ corresponds to the TSPT state with Δ*T* in the L_1_S_2_ and S_1_L_2_ paths. Moreover, $G_{TSPT}^{(1)} (t_{L1} ,\,\,t_{S2} )$ and $G_{TSPT}^{(1)} (t_{S1} ,\,\,t_{L2} )$ describe the effects of the TSPT states in Franson-type interferometry, as in the case shown in Fig. 1b.

Additionally, the time difference $\tau = t_{1} - t_{2}$ between the detection times of both SPDs is determined by the path-length differences of the four possible scenarios concerning the short and long paths. The first-order correlation function *g*^(1)^(*τ*) is applicable to the two cases of the S_1_S_2_ and L_1_L_2_ paths whereas $g_{TSPT}^{(1)} (\tau )$ corresponds to the TSPT state with Δ*T* in the cases of L_1_S_2_ and S_1_L_2_. The thermal light in the Doppler-broadened atomic system can be expressed as $g^{(1)} (\tau ) = g_{TPST}^{(1)} (\tau ) = \exp \left[ { - \pi (\tau /\tau_{c} )^{2} } \right]$, where $\tau_{c}$ denotes the coherence time of the thermal light. Therefore, the normalized second-order correlation function $g^{(2)} (\tau )$ in Franson-type interferometry can be described as

4 $$ g^{(2)} (\tau ) = 1 + \frac{\eta }{4}\left\{ {\begin{array}{*{20}l} {\left| {g^{(1)} (\tau - \Delta T)} \right|^{2} } \hfill \\ { + 2\left| {g^{(1)} (\tau )} \right|^{2} + \left( {e^{{i(\phi_{L} - \phi_{S} )}} + e^{{ - i(\phi_{L} - \phi_{S} )}} } \right)\left| {g_{TPST}^{(1)} (\tau )} \right|^{2} } \hfill \\ { + \left| {g^{(1)} (\tau + \Delta T)} \right|^{2} } \hfill \\ \end{array} } \right\}, $$

where the phase factors $\phi_{L}$ and $\phi_{S}$ can be expressed as $\phi_{L} = \frac{\omega }{c}(L_{1} - L_{2} )$ and $\phi_{S} = \frac{\omega }{c}(S_{1} - S_{2} )$, respectively. Here, *ω* and *c* denote the angular frequency of the signal photon and the speed of light in vacuum, respectively. In the case of time–energy entangled photon pairs, the second-order correlation function of the photon pair is proportional to the temporal two-photon waveform shapes in the Franson interferometer^7^. Therefore, $g^{(2)} (\tau )$ in Eq. (4) represents the temporal waveform of the thermal light in Franson-type interferometry (see Supplementary Note 1 for further details ^20^).

While the original Franson interference is related to the second-order cross-correlation function with entangled photon pairs, the term for SOI in this experiment is related to the second-order self-correlation function of the TSPT state of the thermal light. In particular, the observed interference is contributed to the temporally separated thermal photons, with the separation corresponding to the time difference of the long-short (L_1_S_1_ and L_2_S_2_) paths of both the unbalanced interferometers.

**Figure 2:**
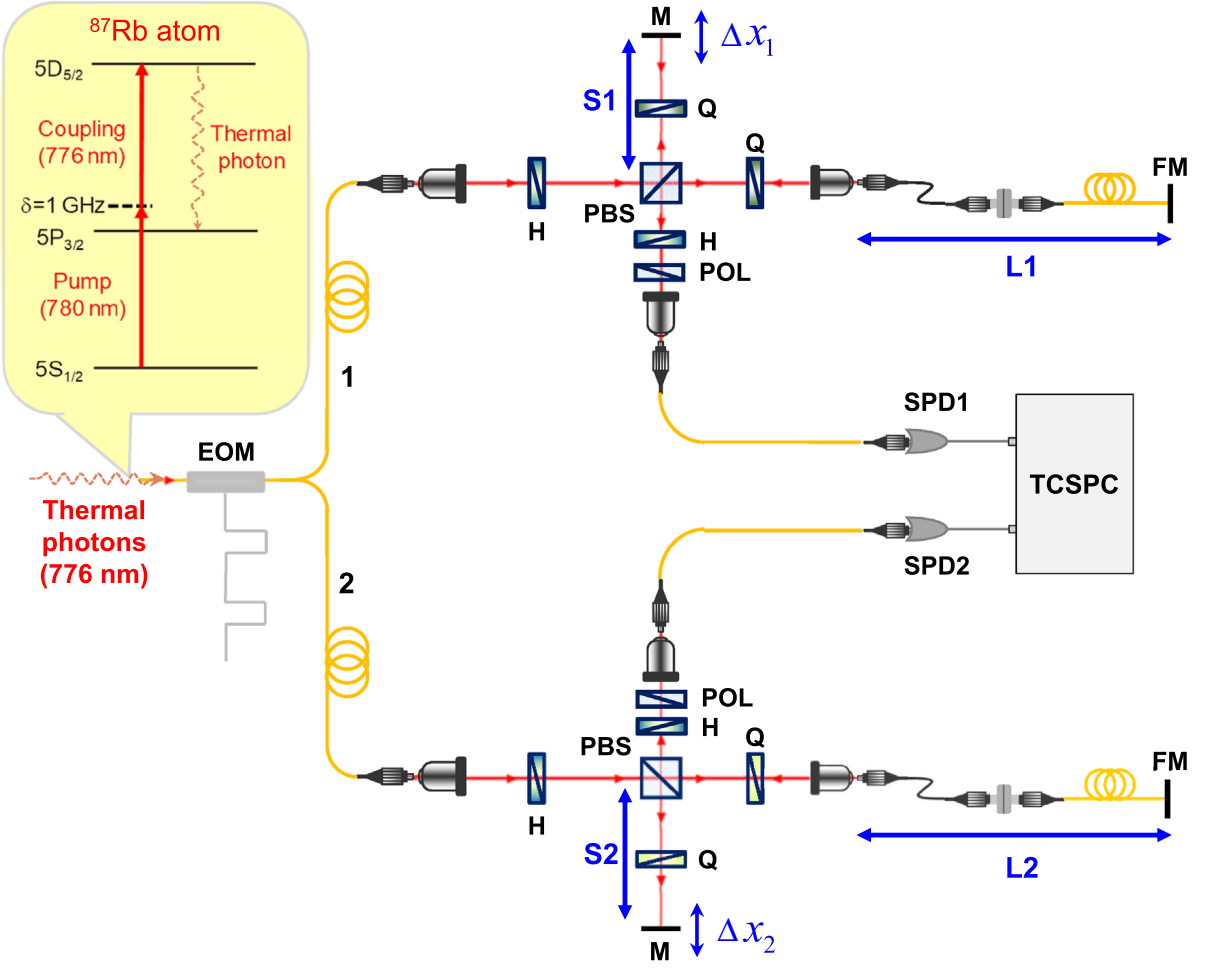
Experimental setup for Franson-type interference with thermal light. SOI obtained using unbalanced Michelson interferometers with large path difference (EOM: electro-optic modulator; H: half-wave plate; Q: quarter-wave plate; M: mirror; POL: polarizer; PBS: polarizing beam splitter; FM: fiber mirror; SPD: single-photon detector; TCSPC: time-correlated single-photon counting module). Numerically calculated phase-matching function values as a function of the propagating angle (*θ*) of the generated FWM for various tilt angles (*θ*_d_) of the driving laser in the four-wave mixing process using a Doppler-broadened cascade-type atomic system.

The thermal light used in our experiment was obtained from a Doppler-broadened cascade-type atomic ensemble based on the 5S_1/2_–5P_3/2_–5D_5/2_ transition of ^87^Rb^21^. The properties of our real thermal light from the warm Rb atoms are not only bright but also long-coherence length. As shown in Fig. 2, the Franson-type interferometry setup consists of two unbalanced Michelson interferometers (UMIs) with a large path difference. The long paths (L_1_ and L_2_) of both UMIs are 2-m long, including an optical fiber mirror.

To confirm the role of the TSPT state of thermal photons in Franson-type interferometry, we modulated the CW-mode thermal photons to a square form with an electro-optic modulator (EOM), as shown in Fig. 2. With this setup, we can determine whether the TSPT state is present or absent by adjusting the modulation period of the EOM. In particular, when the period of the modulation input signal is double the Δ*T* value, the TSPT state in Fig. 1b is absent. In this experimental scenario, we consider the observation of the SOI fringe with thermal photons as a function of path-length differences Δ*x*_1_ or Δ*x*_2_ of the short arms of both UMIs.

## Experimental results and discussion

**Figure 3:**
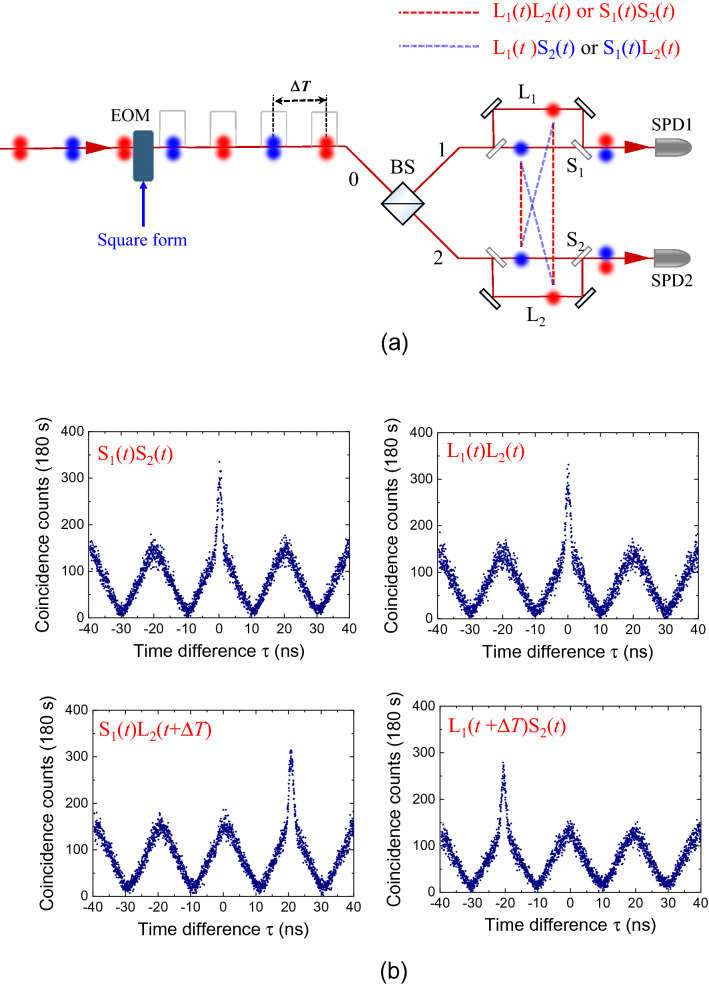
Square-modulated thermal photons with period of Δ*T*. (**a**) Experimental schematic for measurement of the second-order self-correlation functions of the square-modulated thermal photons with period of 20 ns. (**b**) Coincidence events in four cases of short-short (S_1_S_2_), long-long (L_1_L_2_), long-short (L_1_S_2_), and short-long (S_1_L_2_) paths.

First, we consider the TSPT state ($\left| \Psi \right\rangle {|}_{{{\text{TSPT}}}}$) with Δ*T* mentioned in Eq. (1) (as shown in the experimental schematic of Fig. 3a), when the period of the square modulation corresponds to Δ*T* = 20 ns. To understand the characteristics of the square-modulated thermal photons in the Franson-type interferometry, we measured the second-order self-correlation functions of the square-modulated thermal photons in the four cases of the S_1_S_2_, L_1_L_2_, L_1_S_2_, and S_1_L_2_ paths by blocking the optical paths of both UMIs in Fig. 2; the results are shown in Fig. 3b. These results correspond to those of the phase-independent HBT experiment. Here, the triangular form of the coincidence background is due to the convolution of the input square modulation of thermal photons, and its period corresponds to that of the square modulation.

In the cases of S_1_S_2_ and L_1_L_2_ (corresponding to the red dashed lines in Fig. 3a), the coincidence count at τ = 0 is maximum and $g^{(2)} (0)$ is estimated to be 1.76(2), which corresponds to the bunched-light $g^{(2)} (0)$ value of the CW-mode thermal photons (see Supplementary Note 2 for further details^20^). In addition, in the cases of S_1_L_2_ and L_1_S_2_, we note that $g^{(2)} (\Delta T)$ and $g^{(2)} ( - \Delta T)$ exhibit their maximum values at the time delay between the short and long paths. In particular, both photons separated by Δ*T* before the BS can be coincidently detected at τ = 0 in the cases of L_1_S_2_ or S_1_L_2_ (blue dashed lines in Fig. 3a); however, we note that there is no correlation between the two temporally separated photons (Fig. 3b). Because of the breaking of the optical paths in the Franson-type interferometer, the which-way information of both photons is determined, and we cannot consider the TSPT state with Δ*T* in the results shown in Fig. 3b.

**Figure 4:**
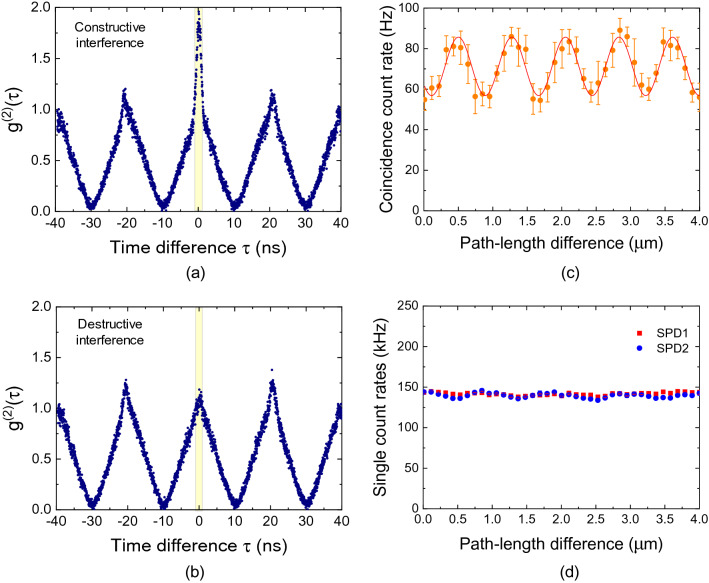
Temporal waveforms of thermal light obtained with Franson-type interferometer. Experimental (blue dots) and theoretical (red curves) results for (**a**) the constructive SOI condition of $\phi_{L} - \phi_{S} =$ 0, (**b**) destructive SOI condition of $\phi_{L} - \phi_{S} =$ π. (**c**) SOI fringe of the thermal light as a function of the path-length difference between both unbalanced Michelson interferometers (Δ*x*_1_ changed and fixed at Δ*x*_2_ = 0). (**d**) Single count rates of SPD1 (blue circles) and SPD2 (red circles) as a function of the path-length difference.

Next, we consider the four cases in Fig. 3 simultaneously in the Franson-type interferometry. Here, it is obvious to assume that the temporal waveform in the Franson-type interferometry is the sum of the four results shown in Fig. 3b. However, interestingly, we observe the interference effect from the temporal waveforms of thermal photons in the Franson-type interferometry, as shown in Fig. 4a, b, under constructive and destructive interference conditions, respectively. Under the constructive interference condition (Fig. 4a), we note that the temporal waveforms of thermal light consist of two side peaks due to the long-short (L_1_S_2_ and L_2_S_1_) path mismatch of the UMIs and the central peak at τ = 0. Importantly, the central peak for constructive interference in Fig. 4a is not the sum of the self-correlation results shown in Fig. 3b. The magnitude of the central peak is four times that of both side peaks. Next, when Δ*x*_1_ of the short arm of the UMI (Fig. 2) is varied, we observe the magnitude variation of the central peak. In Fig. 4b, the value of $g^{(2)} (0)$ at τ = 0 is close to the normalized value of 1, corresponding to destructive interference. Therefore, to understand this phase-sensitive interference of second-order self-correlation in the Franson-type interferometry, we should consider the indistinguishable events of the TSPT state with Δ*T*, as shown in Fig. 3a.

The $g^{(2)} (0)$ term in Eq. (4) can be simply expressed as

5 $$ g^{(2)} (0)\, = 1 + \frac{\eta }{2}\left[ {1 + \cos \left( {\frac{{\omega_{0} }}{c}(L_{1} - L_{2} ) - \frac{{\omega_{0} }}{c}(S_{1} - S_{2} )} \right)} \right]. $$

Sinusoidal SOI fringes can be obtained according to the path-length difference between the long (L_1_, L_2_) and short (S_1_, S_2_) arms of both UMIs.

When we measure the coincidence counting of the SPD1 and SPD2 with a 2.5-ns temporal window (corresponding to the yellow box shown in Fig. 4a,b), we can observe the sinusoidal SOI fringe of the thermal photon, as shown in Fig. 4c. In our experiment, we varied Δ*x*_1_ or Δ*x*_2_ of the short arms by using the translator stages to investigate the SOI fringe variation as a function of the path-length difference of both UMIs.

Here, the visibility of sinusoidal SOI fringe in Fig. 4c is estimated to be 22%. We note that the visibility of the SOI fringe of thermal photon is limited by the time jitter of the employed SPDs and the temporal window for coincidence counting (see Supplementary Note 3 for further details^20^). The maximum and minimum values of the sinusoidal SOI fringe in Fig. 4c correspond to the temporal waveforms of the thermal light in Fig. 4a,b, respectively. Simultaneously, we analyzed the single count rates of the SPD1 and SPD2, as shown in Fig. 4d. We cannot see the sinusoidal interference fringe as a function of the path-length difference of UMIs because the long-short path-length difference of UMI is 10 times longer than the 0.5-m coherence length of thermal photon.

**Figure 5:**
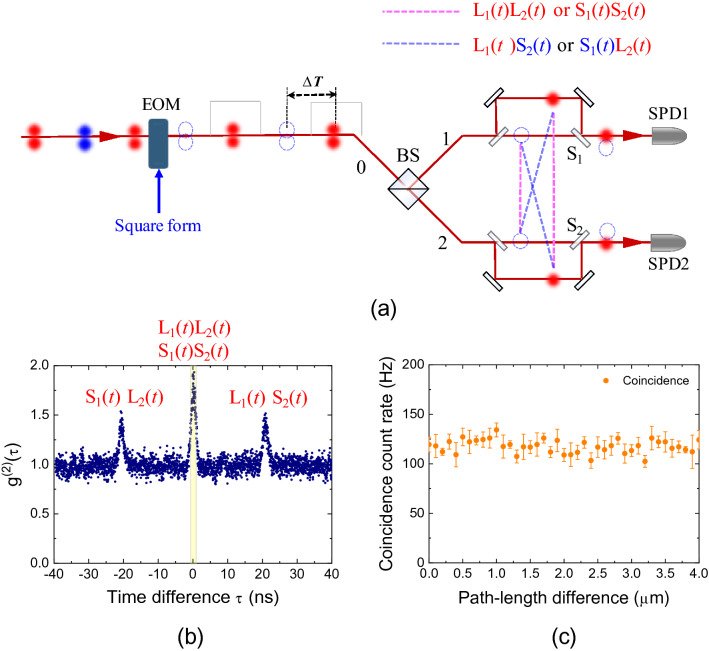
Absence of TSPT state with Δ*T* in Franson-type interferometry. (**a**) Possible coincidence events of both SPDs at τ = 0 with the switching off of the photons (blue circles) after Δ*T* from the instant of switching on of the photons (red circles). (**b**) Temporal waveform of thermal photons in Franson-type interferometer without the TSPT state with Δ*T*. (**c**) Coincidence count rate as a function of the path-length difference (Δ*x*_1_ or Δ*x*_2_).

Next, we “lift” or “break” the TSPT state with Δ*T* using optical switching with the EOM. When the period of the square modulation is set to 2Δ*T* = 40 ns, we can switch off the photons (blue circles) after Δ*T* from the instant of the switching on of the photons (red circles), as shown in Fig. 5a. In this experimental scenario, we cannot consider $\left| \Psi \right\rangle {|}_{{{\text{TSPT}}}}$ with Δ*T* expressed in Eq. (1). In this case, the $\left| {g_{TSPT}^{(1)} (\tau )} \right|^{2}$ term in Eq. (4) is absent. The $g^{(2)} (\tau )$ term in Franson-type interferometry is independent of the phase difference, as can be inferred from Fig. 5b. In our study, the $g^{(2)} (0)$ value was estimated to be 1.75(2), corresponding to that for the constructive interference in Fig. 4a. We note here that the magnitude of the central peak is twice that of both side peaks. Unlike the temporal waveforms shown in Fig. 4a,b, the square modulation of the thermal photons, corresponding to the background in Fig. 5b, does not afford a triangular shape. This is because the triangular form of the coincidence background is out of phase among the second-order self-correlation functions of the square-modulated thermal photons in the four cases (S_1_S_2_, L_1_L_2_, L_1_S_2_, and S_1_L_2_) (see Supplementary Note 4 for further details^20^).

When the detection time difference *τ* is equal to zero, Eq. (5) can be expressed as $g^{(2)} (0)\, = 1 + \frac{\eta }{2}$ for the two cases of short-short (S_1_S_2_) and long-long (L_1_L_2_) paths, where the thermal fraction coefficient *η* is estimated to be 0.75. In Fig. 5c, we cannot observe any sinusoidal SOI fringes as a function of the path-length difference. Therefore, the SOI of thermal photons in Franson-type interferometry is related to the intrinsic indistinguishability of the TSPT states of thermal photons, which is independent of the time delay between the temporally separated photons.

## Conclusion

In conclusion, we have determined the coherence length of TSPT states of thermal photons emitted from a warm atomic ensemble in Franson-type interferometry. Using the novel method of square-modulated thermal photons, we could actively choose whether or not to erase which-path information of the TSPT states of thermal photons in Franson-type interferometry. We confirmed that the cause of SOI of thermal photons in Franson-type interferometry is the intrinsic indistinguishability of the TSPT states of thermal photons. Because the indistinguishability of the TSPT state in the Franson-type interference is independent of the temporal separation of the thermal photons in the TSPT states, the coherence length of the TSPT state of the thermal photons in a Franson-type interferometry is unlimited.

## Supplementary Information

Supplementary Information.
