# Supplementary material for: Indistinguishability of temporally separated pairwise two-photon state of thermal photons in Franson-type interferometry
Source: Sci Rep. 2022 Mar 31;12:5456. doi: 10.1038/s41598-022-09516-y (PMC8971477; doi:10.1038/s41598-022-09516-y)
Supplement: Supplementary file 1 — Supplementary Information. [file 41598_2022_9516_MOESM1_ESM.docx]

**Supplementary Information**

Jiho Park, Heonoh Kim, and Han Seb Moon

*Department of Physics, Pusan National University, Geumjeong-Gu, Busan 46241, Korea*

**Note 1. Temporal waveforms of CW-mode thermal light** **in Franson-type interferometer**

The spectral features of the three peaks are identical to the self-correlation function curve of the thermal light from the warm atomic ensemble. Furthermore, the central peak for the constructive interference case in Fig. S1(a) exactly corresponds to the sum of the self-correlation function curve and the second-order correlation function curve of the temporally separated pairwise two-photon (TSPT) state of the thermal light. The magnitude of the central peak is four times that of both side peaks. In Fig. S1(b), the coincidence counts at τ = 0 exhibit destructive interference. Here, the detection time difference (Δ*T*) between the short and long paths is 16 ns, corresponding to the optical path lengths of both the long arms (*L*_1_ = *L*_2_).

We calculated the temporal waveform shapes using Eq. (4) of our manuscript, and the calculated values are indicated by the red curves in Figs. S1(a) and S1(b). The parameter settings for the theoretical curve are *η* = 0.75, Δ*T* = 16 ns, and $\tau_{c}$*=* 2.0 ns. Upon comparing the experimental and theoretical temporal waveform shapes in both unbalanced Michelson interferometers (UMIs), we note that the theoretical results closely agree with the experimental results. Interestingly, the temporal waveforms of the thermal light in the two spatially separated UMIs are similar to the temporal two-photon waveforms of time–energy entangled photon pairs for Franson interferometry with twin nonlocal unbalanced interferometers [1].

**Figure S1. Temporal waveforms of thermal light obtained in Franson-type interferometer.** (a) Experimental (blue dots) and theoretical (red curves) results for (a) constructive TPI condition of $\varphi_{L}-\varphi_{S}=$0, (b) destructive TPI condition of $\varphi_{L}-\varphi_{S}=$π.

**Note 2. Limitation of maximum *g*^(2)^(0) value of thermal light from Doppler-broadened warm atomic vapor**

Herein, we discuss in detail the experimental limitations of the maximum $g^{(2)}(0)$ value of the thermal light from Doppler-broadened ^87^Rb atoms. To analyze the experimental results, we considered the time jitter of single-photon detectors (SPDs) in the setup. Our SPD time jitter of ~0.4 ns is less than the $g^{(2)}(\tau)$ full-width at half-maximum (FWHM) value of 2.0 ns owing to the Doppler broadening in the atomic vapor cell.

First, we consider the convolution of the $g^{(2)}(\tau)$ function and the time jitter (0.4 ns) of the employed SPD, as shown in Fig. S2 [2−4]. Considering the detector time resolution *R*(*t*-*τ*) as a Gaussian function, we can express the convolution integral as

$(g^{(2)}(\tau)\otimes R)(t)=\int g^{(2)}(\tau)\times R(t-\tau)d\tau$. (S1)

The red curve in Fig. S2 indicates the calculated convolution of the $g^{(2)}(\tau)$ function with a detector resolution of 0.4 ns. The calculated result is in close agreement with the measured $g^{(2)}(\tau)$ function. Moreover, the calculated $g^{(2)}(0)$ value is identical to the measured $g^{(2)}(0)$ value of 1.75. When the time jitter of the SPD is ignored, the calculated $g^{(2)}(0)$ value is substantially closer to 2, as indicated by the blue curve in Fig. S1. The main cause of the discrepancy in the case of our experiment is the dominance of the time jitter of the employed SPDs over the finite spatial coherence.

Figure S2. Simulation of normalized second-order self-correlation function *g*^(2)^(*τ*) of thermal light from Doppler-broadened warm atomic vapor. Convolution between $g^{(2)}(\tau)$ and the 0.4 ns single-photon detector (SPD) time jitter (red line) and calculated $g^{(2)}(\tau)$ spectrum without SPD time jitter (blue line).

**Note 3. Maximum visibility of sinusoidal two-photon interference with thermal photons considering coincidence window**

Considering the time jitter of the employed SPDs and the coincidence window (*T*_w_), we can estimate the maximum visibility of the sinusoidal TPI fringe. Figure S3(a) shows the temporal statistical spectrum of the signal photons obtained using the HBT experiment, corresponding to the normalized second-order correlation function $g^{(2)}(\tau)$. The $g^{(2)}(0)$ value is estimated to be ~1.75 for the SPD time jitter of 0.4 ns. The coincidence count rate represents the integrated coincidence count with *T*_w_ = 2.5 ns in the $g^{(2)}(\tau)$ spectrum of the thermal light, as shown in Fig. S3(a). In the figure, area A (red) denotes the reference (background) of $g^{(2)}(\tau)$ in the *T*_w_ range of 2.5 ns and area B (blue) denotes the $g^{(2)}(\tau)$ Gaussian function with FWHM = 2 ns corresponding to the intensity correlation with the bunching photons of thermal light. Area A is estimated as *T*_w_ (2.5 ns) × 1 (normalized coincidence count) = 2.5, and area B can be expressed in the form

$\int_{-T_{w}/2}^{T_{w}/2} (g^{(2)}(\tau)-1) d\tau=\int_{-T_{w}/2}^{T_{w}/2} \eta\cdot e^{-\pi(\tau/\tau_{c})^{2}} d\tau$, (S2)

where *η* represents the thermal fraction coefficient and $\tau_{c}$ the coherence time of the thermal light. For the parameters of *η* = 0.75, *T*_w_ = 2.5 ns, and $\tau_{c}$*=* 2.0 ns, area B denotes the area under the curve, and it is calculated to be 1.4.

As shown in Fig. S3(b), the maximum TPI fringe value for constructive interference corresponds to a total area (A + B) of 3.9, and the minimum TPI fringe value for destructive interference corresponds to an A-area value of 2.5. Therefore, the visibility of the TPI fringes shown in Fig. S3(b) is calculated to be 21.8%.

**Figure S3.** **Calculation of two-photon interference (TPI) visibility with thermal light.** (a) Normalized second-order correlation function of thermal light from Doppler-broadened cascade-type ^87^Rb atoms. (b) TPI fringe of the thermal light as a function of the path-length difference between both unbalanced Michelson interferometers (UMIs).

**Note 4. Second-order self-correlation function of square-modulated thermal photons with period of 2**Δ**T**

When the period of the square modulation is set to 2Δ*T* = 40 ns, we can switch off the photons (blue circles) after Δ*T* from the instant of the switching on of the (red circle) photons, as shown in Fig. S4(a). The temporal waveform was measured via coincidence counting for an accumulation time of 180 s using a time-correlated single-photon counter (TCSPC). Figure S4(b) shows the second-order self-correlation functions of the square-modulated thermal photons in the four cases (S_1_S_2_, L_1_L_2_, L_1_S_2_, and S_1_L_2_). The period of the triangular form of the coincidence background corresponds to that of the input square modulation. We observe the maximum coincidence counting in both cases of S_1_S_2_ and L_1_L_2_ at τ = 0, for the case of S_1_L_2_ at τ = Δ*T*, and for the case of L_1_S_2_ at τ =-Δ*T*. The maximum value of $g^{(2)}(\tau)$ is estimated to be 1.75(2). Interestingly, the phases of the triangular background are out of phase among the four cases (S_1_S_2_, L_1_L_2_, L_1_S_2_, and S_1_L_2_), as shown in Fig. S4(b). Therefore, when we consider the four cases in Fig. S4(b) in Franson-type interferometry, the triangular form of the coincidence background is erased.

**Figure S4.** **Square-modulated thermal photons with period of 2Δ*T*.** (a) Experimental schematic for the measurement of the second-order self-correlation functions of the square-modulated thermal photons with period of 40 ns. (b) Coincidence events in the four cases of short-short (S_1_S_2_), long-long (L_1_L_2_), long-short (L_1_S_2_), and short-long (S_1_L_2_) paths.

**References**

[1] J. Park, D. Kim, H. Kim, and H. S. Moon, “High-visibility Franson interference of time–energy entangled photon pairs from warm atomic ensemble,” Opt. Lett. **44**(15), 3681-3684 (2019).

[2] J. Mika, L. Podhora, L. Lachman, P. Obšil, J. Hloušek, M. Ježek, R. Filip, and L. Slodička, “Generation of ideal thermal light in warm atomic vapor,” New J. Phys. **20**, 093002 (2018).

[3] A. Dussaux, T. Passerat de Silans, W. Guerin, O. Alibart, S. Tanzilli, F. Vakili, and R. Kaiser, “Temporal intensity correlation of light scattered by a hot atomic vapor,” Phys. Rev. A **93**, 043826 (2016).

[4] J. Park, T. Jeong, and H. S. Moon, “Temporal intensity correlation of bunched light from a warm atomic vapor with a ladder-type two photon transition,” Sci. Rep. **8**, 10981 (2018).
